# The shaky foundations of large language models and foundation models for electronic health records

**DOI:** 10.1038/s41746-023-00879-8

**Published:** 2023-07-29

**Authors:** Michael Wornow, Yizhe Xu, Rahul Thapa, Birju Patel, Ethan Steinberg, Scott Fleming, Michael A. Pfeffer, Jason Fries, Nigam H. Shah

**Affiliations:** 1 Department of Computer Science, Stanford University, Stanford, CA USA; 2 Center for Biomedical Informatics Research, Stanford University School of Medicine, Stanford, CA USA; 3 Technology and Digital Services, Stanford Health Care, Palo Alto, CA USA; 4 Department of Medicine, Stanford University School of Medicine, Stanford, CA USA; 5 Clinical Excellence Research Center, Stanford University School of Medicine, Stanford, CA USA

**Keywords:** Machine learning, Computational platforms and environments, Predictive medicine, Data mining, Statistical methods

## Abstract

The success of foundation models such as ChatGPT and AlphaFold has spurred significant interest in building similar models for electronic medical records (EMRs) to improve patient care and hospital operations. However, recent hype has obscured critical gaps in our understanding of these models’ capabilities. In this narrative review, we examine 84 foundation models trained on non-imaging EMR data (i.e., clinical text and/or structured data) and create a taxonomy delineating their architectures, training data, and potential use cases. We find that most models are trained on small, narrowly-scoped clinical datasets (e.g., MIMIC-III) or broad, public biomedical corpora (e.g., PubMed) and are evaluated on tasks that do not provide meaningful insights on their usefulness to health systems. Considering these findings, we propose an improved evaluation framework for measuring the benefits of clinical foundation models that is more closely grounded to metrics that matter in healthcare.

## Introduction

Foundation models (FMs) are machine learning models capable of performing many different tasks after being trained on large, typically unlabeled datasets^1^. FMs represent a paradigm shift in how machine learning (ML) models are developed—rather than developing a bespoke model for each specific use case (as was done traditionally), a single FM can instead be reused across a broad range of downstream tasks with minimal adaptation or retraining needed per task. FMs have received significant attention given their impressive range of capabilities across multiple domains, from text generation^2^ and video editing^3^ to protein folding^4^ and robotics^5^.

One of the most popular FMs has been OpenAI’s ChatGPT, which surpassed 100 million users within two months of release^6^. ChatGPT is a large language model (LLM), a type of FM which ingests text and outputs text in response. Though ChatGPT was trained to simply predict the next word in a sentence—it is basically an advanced autocomplete— incredible capabilities “emerged” from this training setup which allow the model to perform a wide variety of complex tasks involving language^7^. Physicians were quick to apply the model to pass medical licensing exams^8–11^, simplify radiology reports^12^, and write research articles^13^. In addition to text, FMs built on structured EMR data have shown the ability to predict the risk of 30-day readmission^14^, select future treatments^15^, and diagnose rare diseases^16^.

The breakneck progress of AI over the past year has made it difficult for healthcare technology professionals and decision-makers to accurately assess the strengths and limitations of these innovations for clinical applications. Beyond short demos being shared on social media, there is little systematic examination of what the best use cases for production-grade clinical FMs are, or how healthcare organizations should weigh their benefits against their substantial risks^1,17–19^. Clinical FMs lack the shared evaluation frameworks and datasets^20^ that have underpinned progress in other fields, such as natural language processing (NLP) and computer vision^21^. This makes it difficult to quantify and compare these models’ capabilities.

If we believe that FMs can help both providers and patients^22^, then rigorous evaluations must be conducted to test these beliefs. In this review, we uncover notable limitations in how clinical FMs are evaluated and a large disconnect between their evaluation regimes and assumed clinical value. While adopting FMs into healthcare has immense potential^23^, until we know how to evaluate whether these models are useful, fair, and reliable, it is difficult to justify their use in clinical practice. Inspired by recent efforts to holistically evaluate LLMs trained on non-clinical text for a range of capabilities beyond accuracy^24^, we believe that a similar approach is necessary to tie the evaluation of FMs writ large with use cases that matter in healthcare.

To clarify these challenges, we reviewed over 80 different clinical FMs built from electronic medical record (EMR) data. We included all models trained on structured (e.g., billing codes, demographics, lab values, and medications) and unstructured (e.g., progress notes, radiology reports, other clinical text) EMR data, but explicitly excluded images, genetics, and wearables to manage the scope of this review. We refer to the combination of structured and unstructured EMR data (excluding images) as simply “EMR data” or “clinical data”^25^. We refer to FMs built on these forms of clinical data as “clinical foundation models” or “clinical FMs.” Our primary contributions are:

1. *To our knowledge, we present the largest review of clinical FMs for structured and unstructured EMR data*. We organize these models into a simple taxonomy to clearly delineate their architectures, training data, capabilities, and public accessibility.
2. *We summarize the currently used evaluation frameworks for clinical FMs and identify their limitations*. We explain why current evaluation tasks provide little evidence for the purported benefits of FMs to a health system.
3. *We propose an improved framework for evaluating clinical FMs*. We advocate for metrics, tasks, and datasets that better capture the presumed value of clinical FMs.

We begin with a brief overview of clinical FMs and define their inputs, outputs, and capabilities in “What are clinical FMs?”. In “Benefits of clinical FMs”, we summarize the primary value propositions of FMs for health systems. In “State of published clinical FMs”, we provide an overview of the training data behind clinical FMs, examine current evaluation regimens and identify their limitations, and propose a framework for improving these evaluations. Finally, we discuss the promise of clinical FMs for solving a diverse range of healthcare problems in Discussion.

## What are clinical FMs?

A foundation model (FM) is a type of machine learning model that has been pre-trained on large amounts of unlabeled data and can be adapted to a broad range of downstream tasks^1^. FMs leverage a training procedure referred to as “pre-training,” in which a “self-supervised” (i.e., no labels are required) learning objective is used to scale learning to immense amounts (i.e., terabytes) of unlabeled data. FMs also typically have significantly more parameters than traditional ML models—sometimes in the hundreds of billions of parameters—which requires significant computational resources to train (i.e., months of time on a supercomputer with hundreds of GPUs)^26^. The significantly larger size of FMs, coupled with their task-agnostic self-supervised learning objective, has sparked a paradigm shift in how ML models are developed, and has resulted in the “emergence” of unprecedented capabilities at sufficient model scale^27^.

Clinical FMs are foundation models built specifically for electronic medical record data. There are two broad categories of clinical FMs: Clinical language models (CLaMs) and Foundation models for EMRs (FEMRs).

### Clinical language models (CLaMs)

**Fig. 1:**
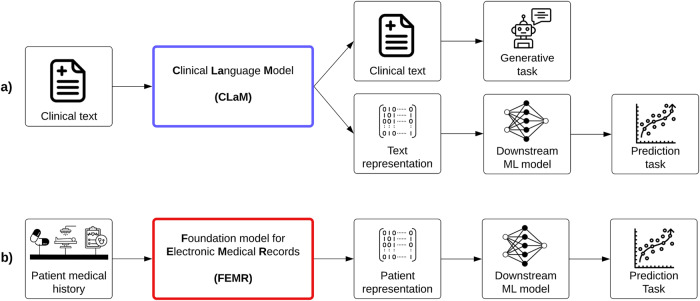
The two types of clinical FMs. Overview of the inputs and outputs of the two main types of clinical FMs. **a** The inputs and outputs of Clinical Language Models (CLaMs). CLaMs ingest clinical text and output either clinical text or a machine-understandable representation of the input text, which can then be used for downstream prediction tasks. **b** The inputs and outputs of Foundation models for Electronic Medical Records (FEMRs). FEMRs ingest a patient’s medical history—which is simply a sequence of medical events with some temporal ordering—and output a machine-understandable representation of the patient, which can then be used for downstream prediction tasks.

The first category of FMs are clinical l*a*nguage models, or CLaMs, which are a subtype of large language models (LLMs). As shown in Fig. 1a, the unique attribute that separates CLaMs from general LLMs is their specialization on clinical/biomedical text—CLaMs are primarily trained on, ingest, and output clinical/biomedical text. For example, a CLaM could extract drug names from a doctor’s note^28^, automatically reply to patient questions^29^, summarize medical dialogues^30^, or predict mechanical ventilation needs based on clinical notes^31^.

While general-purpose LLMs (e.g., ChatGPT, Bloom, GPT-4, etc.) trained on text scraped from the Internet can also be useful for clinical tasks, they tend to underperform CLaMs on domain-specific tasks^32,33^, and thus we exclude them from this discussion. However, the conclusions from this review should also readily apply to these general-purpose models, as they suffer from the same limitations that we describe for CLaMs.

### Foundation models for electronic medical records (FEMRs)

The second class of clinical FMs are foundation models for electronic medical records (FEMRs). These models are trained on the entire timeline of events in a patient’s medical history. Given a patient’s EMR as input, a FEMR will output not clinical text but rather a machine-understandable “representation” for that patient, as shown in Fig. 1b. This representation —also referred to as a “patient embedding”—is typically a fixed-length, high-dimensional vector which condenses large amounts of patient information^34^. A patient’s representation can then be used as input to any number of downstream models for different tasks. These downstream models (built on the “foundation” of FEMR representations) tend to be more accurate and robust than traditional machine learning (ML) models on clinically relevant tasks, such as predicting 30-day readmission or long length-of-stay^35^.

The input to a FEMR can include many aspects of a patient’s medical history, such as structured codes, lab values, claims, and clinical text. In practice, however, FEMRs are typically limited to the single modality of structured codes, as discussed in “State of published clinical FMs”.

Though CLaMs and FEMRs have remained fairly separate over the past several years, we note that the distinction between these two lines of work is becoming increasingly blurred as the next generation of foundation models for EMRs becomes more expressive and multimodal in nature.

### Benefits of clinical FMs

Given the excitement around FMs in healthcare^23,36–41^, we summarize their primary value propositions over traditional ML methods. These advantages could all be highly valuable to a health system. Thus, it is essential that our evaluation tasks, datasets, and metrics provide accurate assessments of these purported benefits.

1. **Clinical FMs have better predictive performance.** By using larger training datasets and more model parameters, FMs can achieve better predictive performance (e.g., higher sensitivity and specificity on classification tasks) than traditional ML models^34^.
2. **Clinical FMs require less labeled data (“improved sample efficiency”).** FMs enable superior model performance using fewer labeled data via “transfer learning”^42^. The core idea behind transfer learning is to first “pre-train” a model on large amounts of non-task-specific (and often unlabeled) data to teach the model general patterns. Then, the model is “fine-tuned” (i.e., continued to be trained) on a smaller dataset specific to the desired task. For example, a sentiment classification model pre-trained on the raw text of Wikipedia before being fine-tuned on a labeled dataset of 100 Tweets will outperform models solely trained on the smaller task-specific dataset of Tweets^42^. Additionally, some FMs can be directly applied to novel tasks without any additional fine-tuning via “zero-shot” or “few-shot” learning. In zero-shot learning, a model learns an entirely new task without being given any specific examples for that task—in other words, the model is given zero examples from which to learn and must instead rely on its general reasoning capabilities to complete the desired task. Similarly, in few-shot learning, the model is only provided with a few examples (typically less than 64) from which to learn. Zero/few-shot learning are particularly powerful capabilities, as they enable FMs to rapidly adapt to new tasks without the need for large, task-specific labeled datasets. Thus, by learning representations that are useful for many downstream tasks via self-supervised pre-training, FMs can greatly reduce the cost of developing ML models for a particular task.
3. **Clinical FMs enable simpler and cheaper model deployment.** After an FM is trained, it can help to decrease the time, talent, and resources required to build subsequent ML models by serving as the figurative “foundation” upon which these subsequent applications are built^1^. Numerous companies have already commercialized this “ML-as-a-Service” approach, in which a centralized FM is made available to end-users via a simple API^43^. A similar approach could work in healthcare, wherein a clinical FM allows informaticians to integrate AI-related capabilities into applications while avoiding the expensive data ingestion, preprocessing, model training, and deployment steps in a typical ML pipeline^44^.
4. **Clinical FMs exhibit “emergent” capabilities that enable new clinical applications.** The large number of parameters in FMs has resulted in a phenomenon known as “emergence,” in which previously intractable problems become tractable at sufficient model scale^7^. For example, CLaMs can now write coherent insurance appeals in ways thought impossible only a couple of years ago^45^, while FEMRs can generate compact patient representations that enable time-to-event modeling of hundreds of outcomes simultaneously^46^.
5. **Clinical FMs can more effectively handle multimodal data.** FMs can be designed to accept a wide range of data modalities (e.g., structured codes, lab values, clinical text, images, speech patterns, etc.) as inputs and incorporate them into a single unified representation^47^. Substantial prior work has shown that these models’ large parameter counts and dataset sizes enable them to effectively model disparate modalities in the same shared latent space, thereby deriving richer representations for each modality than possible with unimodal models^48–51^. This is especially useful in medicine, given the many types of data produced by patients^52^. For example, a model might simultaneously consider an MRI scan, vital signs, and progress notes when predicting a patient’s optimal treatment^53^.
6. **Clinical FMs offer novel interfaces for human-AI interaction.** Via a technique called “prompting”, a human can input natural language into an LLM and have the model respond in natural language^2^. This enables a two-way conversation between humans and machine, and allows for the decomposition of problems into smaller steps via techniques such as “chain-of-thought” prompting^54^. Prompting generalizes beyond natural language. For example, a FEMR could be prompted with a desired clinical end state (e.g., normal A1C level) to identify which medications should be prescribed to achieve it^55^.

## State of published clinical FMs

We identified 84 distinct clinical FMs published before March 1, 2023. Specifically, we identified 50 CLaMs and 34 FEMRs by following citations from several representative samples of recent work, as well as manual article curation. Given the rapid pace at which this field advances, we do not claim to include every possible model or cover every recent advancement in the clinical FM space, but rather aim at capturing the general narrative direction of the field. We believe the papers that we selected should adequately capture the general themes that would be identified in other types of reviews, as they are representative of the most recent work in the field, and therefore do not make any claims about the systematicity of our search process. We focus exclusively on models that utilize structured and unstructured EMR data (excluding images) to scope this review.

In the following section, we review the training data and public availability of both CLaMs and FEMRs.

### CLaMs

#### Training data

**Fig. 2:**
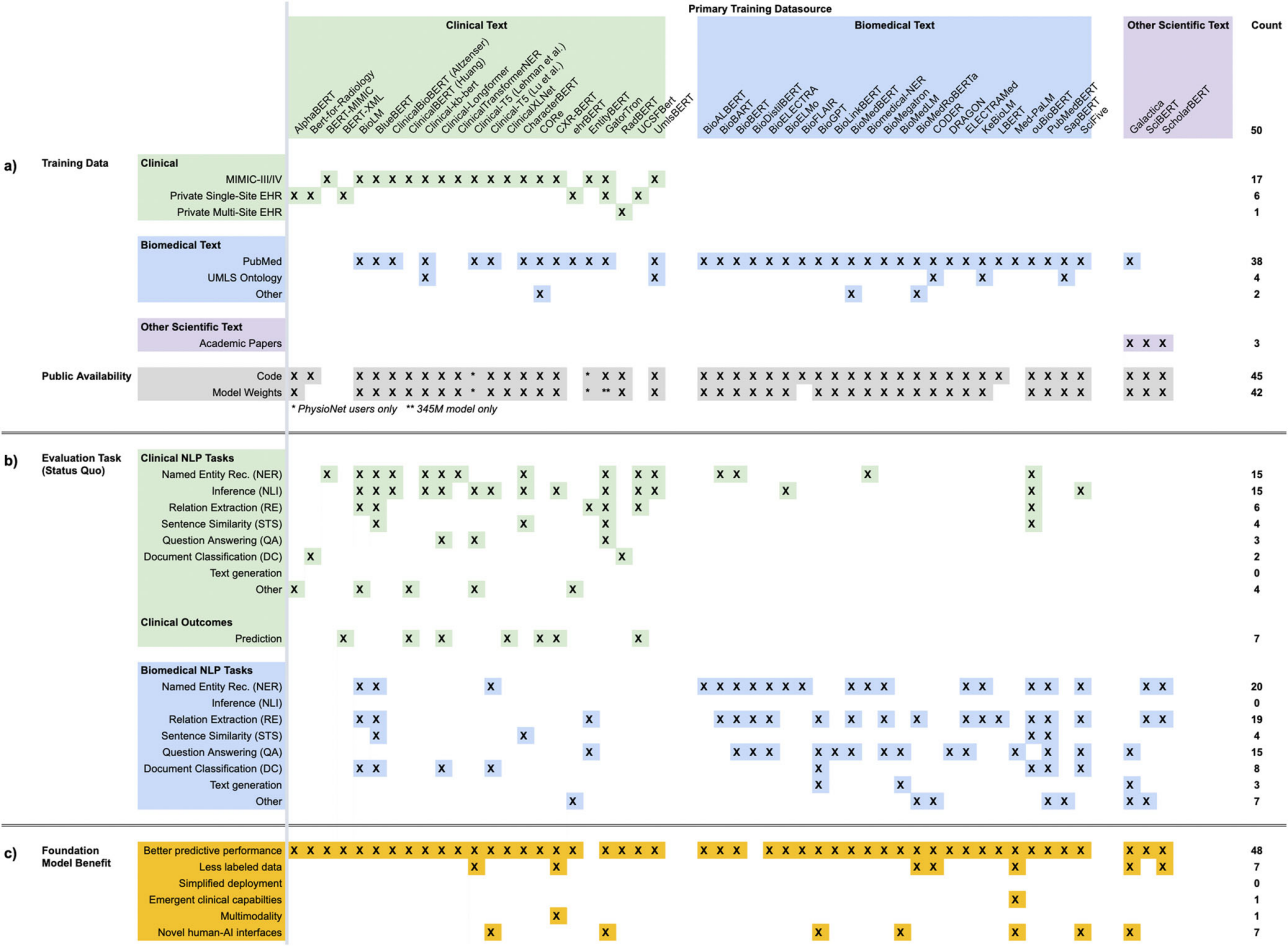
Overview of CLaMs. A summary of CLaMs and how they were trained, evaluated, and published. Each column is a specific CLaM, grouped by the primary type of data they were trained on. Columnwise, the CLaMs primarily trained on clinical text are green (*n* = 23), those trained primarily on biomedical text are blue (*n* = 24), and models trained on general academic text are purple (*n* = 3). The last column is the count of entries in each row. An **X** indicates that the model has that characteristic. An * indicates that a model partially has that characteristic. **a** Training data and public availability of each model. The top rows mark whether a CLaM was trained on a specific dataset, while the bottom-most row records whether a model’s code and weights have been published. Almost all CLaMs have had their model weights published, typically via shared repositories like the HuggingFace Model Hub. **b** Evaluation tasks on which each model was evaluated in its original paper. Green rows are tasks whose data were sourced from clinical text and blue rows are evaluation tasks sourced from biomedical text. The tasks are presented by the way they are commonly organized in the literature. CLaMs primarily trained on clinical text are evaluated on tasks drawn from clinical datasets, while CLaMs primarily trained on biomedical text are almost exclusively evaluated on tasks that contain general biomedical text (i.e., not clinical text). **c** Clinical FM benefits on which each model was evaluated in its original paper. The underlying tasks presented in this section are identical to those in (**b**), but here the tasks are reorganized into six buckets that reflect the six primary FM benefits described in Benefits of clinical FMs. While almost all CLaMs have demonstrated the ability to improve predictive accuracy over traditional ML approaches, there is scant evidence for the other five value propositions of clinical FMs.

CLaMs (Fig. 2a) are primarily trained on either clinical text (i.e., documents written during the course of care delivery) or biomedical text (i.e., publications on biomedical topics). Almost all CLaMs trained on the clinical text used a single database: MIMIC-III, which contains approximately 2 million notes written between 2001–2012 in the ICU of the Beth Israel Deaconess Medical Center^56^. CLaMs trained on biomedical text are virtually always trained on PubMed abstracts and/or full-text articles. While most CLaMs trained on clinical text are also trained on biomedical text, the converse is not true.

#### Model availability

Almost all CLaMs have been made publicly accessible via online model repositories like HuggingFace^57^. Unfortunately, the exceptions are the very CLaMs that seem to have the best performance^58^ —ehrBERT^59^, UCSF-Bert^58^, and GatorTron^60^— as they were trained on private EMR datasets.

#### Takeaways

The high number of CLaMs published over the past several years may lead us to mistake motion for progress. Nearly all CLaMs have been trained on just two datasets -- MIMIC-III and PubMed, which respectively contain about 2 million clinical notes and 16 million abstracts with 5 million full-text publications. Combined, these two datasets contain about 18.5 billion words, which means models trained on them have substantial gaps in completeness (i.e., any scientific knowledge not contained within these corpora) and timeliness (i.e., any new diseases, treatments, or practices discovered after 2012 in the case of MIMIC-III). Empirically, we see that models trained on large-scale EHR data outperform CLaMs trained on shared public datasets across-the-board on out-of-domain data distributions^32,58^.

### FEMRs

#### Training data

**Fig. 3:**
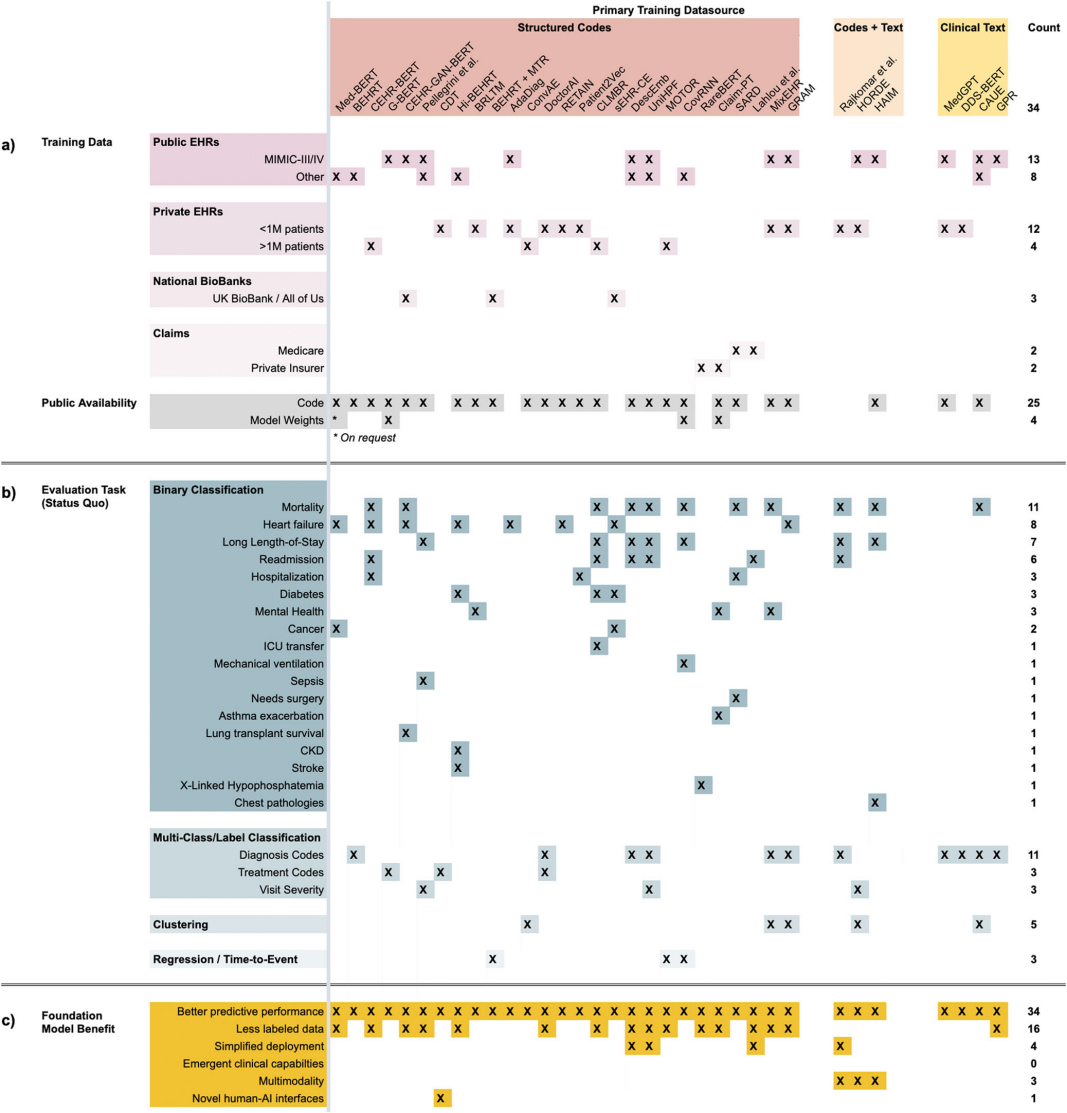
Overview of FEMRs. A summary of FEMRs and how they were trained, evaluated, and published. Each column is a specific FEMR, grouped by the primary type of data they were trained on. Columnwise, the FEMRs primarily trained on structured EMR codes (e.g., billing, medications, etc.) are red (*n* = 27), those trained on both structured codes and clinical text are orange (*n* = 3), and models trained only on clinical text are yellow (*n* = 4). The last column is the count of entries in each row. An **X** indicates that the model has that characteristic. An * indicates that a model partially has that characteristic. **a** Training data and public availability of each model. The top rows mark whether a FEMR was trained on a specific dataset, while the bottom-most row records whether a model’s code and weights have been published. Very few FEMRs have had their model weights published, as they are limited by data privacy concerns and a lack of interoperability between EMR schemas. **b** Evaluation tasks on which each model was evaluated in its original paper. From top to bottom, the evaluation tasks are binary classification, multi-class/label classification, clustering of patients/diseases, and regression tasks like time-to-event. The tasks are presented by the way they are commonly organized in the literature. FEMRs are evaluated on a very broad and sparse set of evaluation tasks—even the same nominal task will often have different definitions across papers. **c** Clinical FM benefits on which each model was evaluated in its original paper. The underlying tasks presented in this section are identical to those in (**b**), but here the tasks are reorganized into six buckets that reflect the six primary FM benefits described in “Benefits of clinical FMs”. While almost all FEMRs have demonstrated the ability to improve predictive accuracy over traditional ML approaches, and a significant number have demonstrated improved sample efficiency, there is scant evidence for the other four value propositions of clinical FMs.

Most FEMRs (Fig. 3a) are trained on either small, publicly available EMR datasets or a single private health system’s EMR database. Again, the most popular public dataset is MIMIC-III, which contains less than 40,000 patients^56^. Other public datasets vary greatly in size, from eICU’s 139,000 patients^61^ to the CPRD’s longitudinal records on 7% of all patients in the UK^62^. Several FEMRs have been trained on insurance claims, which are typically larger in size and more diverse than EMR data but contain less granular information^63^. Examples of claims datasets include Truven Health MarketScan (170 million patients)^64^ and Partners For Kids (1.8 million pediatric patients)^65^. In terms of data modalities, most FEMRs are unimodal as they only consider structured codes (e.g., LOINC, SNOMED, etc.).

#### Model accessibility

FEMRs lack a common mechanism like HuggingFace for distributing models to the research community, as can be seen in the sparsity of the bottom-most row in Fig. 3a compared to the density of the bottom-most row in Fig. 2a. Few FEMRs have had their model weights published, meaning researchers must re-train these models from scratch on local EMR data to verify their performance.

#### Takeaways

The overreliance on structured codes limits the generalizability of FEMRs across health systems that use different EMR systems and coding practices. Some models, such as DescEmb, address this problem by first converting coded data into their textual descriptions, thus detaching the model from the specific codes on which it was trained^66^. An additional limitation of relying on coded data is that it contains inconsistencies and errors^67^, and often provides an incomplete picture of patient state^68^. Some FEMRs have tackled this problem by combining unstructured EHR data (i.e., text) with structured EMR data to boost performance on specific phenotyping and prediction tasks^69,70^. However, the key unsolved challenge of how to publicly share pre-trained FEMRs continues to hinder the field’s progress and precludes the primary value proposition of FMs—namely, being able to build off a pre-trained model.

Next, we considered the common evaluation frameworks for clinical FMs. The common thread between most of these evaluations is that they are relatively straightforward to conduct in an automated fashoin. While these tasks provide diagnostic insights on model behavior, they provide limited insight into the claims of FMs being a “categorically different” technology^71,72^, and offer little evidence for the clinical utility achieved by these models. Taking inspiration from the broader ML community’s push towards Holistic Evaluation of Language Models^24^, we do a critical evaluation of the evaluations currently used to evaluate clinical FMs.

### CLaMs

#### Evaluation of standard tasks and datasets

We collected every evaluation task that a CLaM was evaluated on in its original publication in Fig. 2b, and grouped these tasks as they are commonly reported in the literature. Most CLaMs are being evaluated on traditional NLP-style tasks such as named entity recognition, relation extraction, and document classification on either MIMIC-III (clinical text) or PubMed (biomedical text)^73,74^. Given that clinical text has its own unique structure, grammar, abbreviations, terminology, formatting, and other idiosyncrasies not found in other domains^75^, it is alarming that roughly half of all CLaMs surveyed were not validated on clinical text, and thus may be overestimating their expected performance in a healthcare setting.

When NLP tasks are sourced from clinical text, they can be useful measures of a model’s linguistic capabilities. However, these NLP tasks are greatly limited by their overreliance on the same handful of data sources^74^, small dataset sizes (typically thousands of examples)^74,76^, highly repetitive content^77^, and low coverage of use cases^20^. As a result, strong performance on a clinical NLP task does not provide compelling evidence to a hospital looking to deploy a CLaM— claiming that *“Model A achieves high precision on named entity recognition on 2,000 discharge notes from MIMIC-III*” is very different than “*Model A should be deployed across all of Health System X to identify patients at risk of suicide*”.

#### Evaluation on FM benefits

To illustrate the disconnect between current evaluation tasks and the loftier promises of clinical FMs, we reorganized the rows of evaluation tasks from Fig. 2b—originally presented as they are typically grouped in the literature—along the six primary FM value propositions from “Benefits of Clinical FMs”. The result is Fig. 2c, which identifies which CLaMs were evaluated against any of the six core benefits of clinical FMs. Most CLaMs have only shown evidence for one FM value proposition: improved predictive accuracy on certain tasks. However, there is little evidence supporting the other purported benefits of FMs, such as simplified model deployment or reducing the need for labeled data. For example, while zero- and few-shot prompting techniques have been rigorously studied for general-purpose LLMs as an important method for achieving improved performance, few CLaMs have been evaluated across different prompting strategies and fine-tuning techniques. In other words, there is a gap in our understanding of what CLaMs *can do* versus what CLaMs can do that is valuable to a health system and which traditional ML models cannot do.

### FEMRs

#### Evaluation on standard tasks and datasets

We collected the original tasks on which each FEMR was evaluated in Fig. 3b and bucketed them as they are typically presented in the literature. Evaluation of FEMRs is in an even poorer state than that of CLaMs. While CLaMs benefit from the NLP community’s adoption of standardized task formats, FEMRs lack a similar set of “canonical” evaluations. Instead, FEMRs are evaluated on an extremely sparse set of tasks with little-to-no overlap across publications. This makes it highly non-trivial to compare the performance of different FEMRs.

These tasks are typically grouped by how each task is formulated, e.g., binary classification v. multi-label classification v. regression. The most popular prediction tasks are binary classification tasks such as mortality, heart failure, and long length-of-stay, but even the same nominal task can have widely divergent definitions across papers^78^.

#### Evaluation on FM benefits

We reorganized the rows of evaluation tasks from Fig. 3b along the six primary value propositions of clinical FMs listed in “Benefits of Clinical FMs”. The result is Fig. 3c, which shows that almost all evaluations of FEMRs have been focused on demonstrating their superior predictive accuracy over traditional ML models. Notably, the ability to use less labeled data (i.e., sample efficiency) has been fairly well-documented with FEMRs. However, the other four potential benefits of FMs have gone largely unstudied. And while evaluations of predictive accuracy are straightforward to perform, it is not the sole property of FMs that would justify their adoption by a health system.

**Fig. 4:**
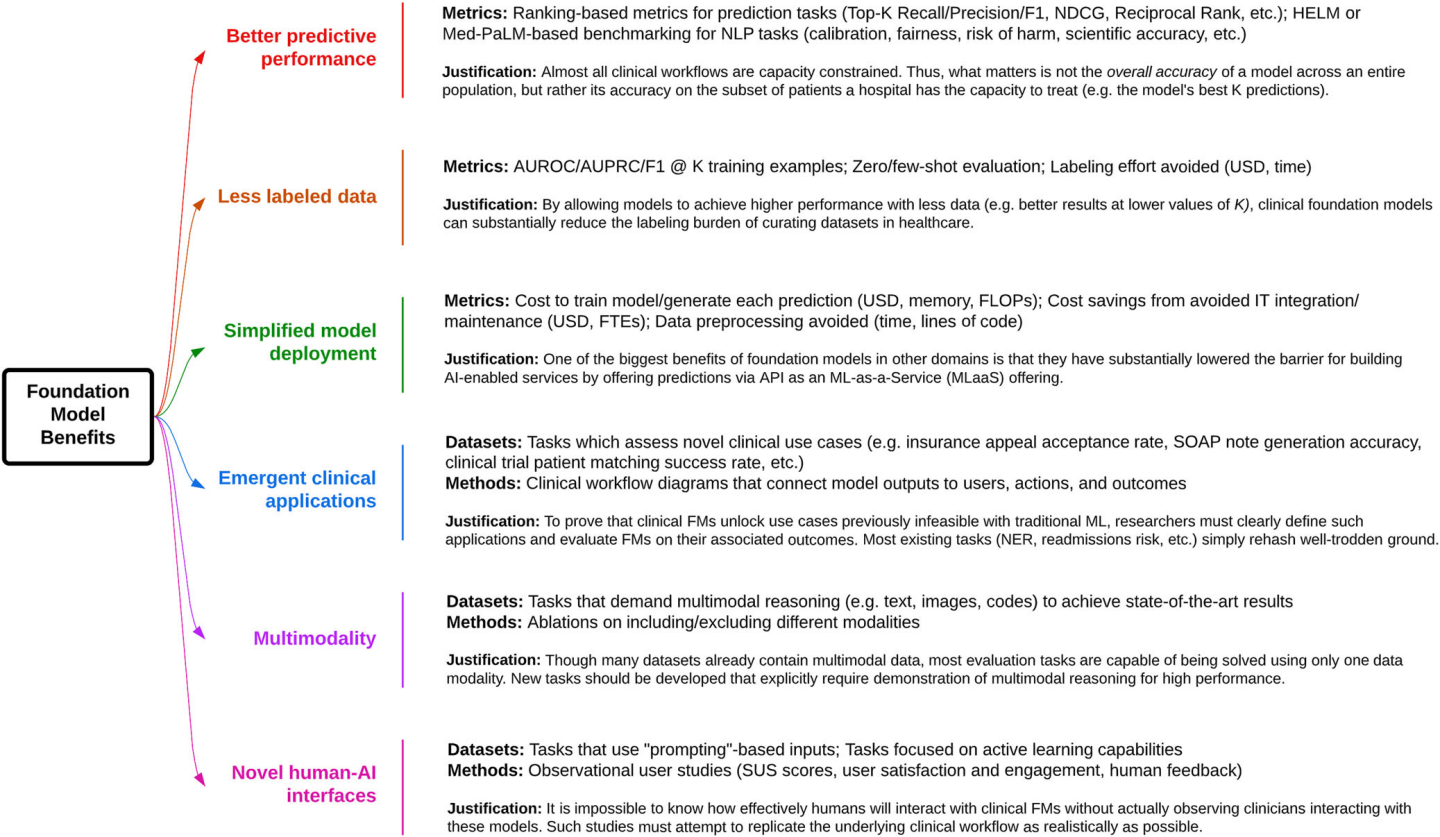
Better evaluations of clinical FMs. Proposals for how to demonstrate the value of CLaMs and FEMRs for achieving the six primary value propositions of FMs to health systems over traditional ML models.

Finally, to better quantify the ability of clinical FMs to achieve the six key benefits of FMs outlined in “Benefits of clinical FMs”, we propose several improved evaluation metrics and tasks in Fig. 4. Our suggestions are by no means comprehensive, but rather meant to spark a further discussion on how to align model evaluation with the demonstration of clinical value.

1. **Better predictive performance:** The most thoroughly studied property of clinical FMs has been their improved predictive performance on classification and regression tasks based on AUROC, AUPRC, F1 Score, and Accuracy. These metrics assume an infinite capacity to act on a model’s predictions. In reality, clinical workflows are capacity constrained—a nursing team may only be able to act on a handful of model predictions per day^79,80^. Thus, a health system should only care about a model’s accuracy on patients for which it has the capacity to intervene. We, therefore, recommend that researchers adopt ranking-based metrics (e.g., Top-K precision/recall/F1, reciprocal ranking, etc.), which are commonly used for recommendation systems^81^. Additionally, we propose examining not just a model’s ability to classify patients correctly but also its calibration across subgroups, fairness, and alignment with clinical best practices^24,29^. The human evaluation may also be necessary in some cases, such as evaluating a CLaM’s ability to accurately generate answers to clinical questions^82^. Traditional NLP metrics such as ROUGE, METEOR, and BLEU—which simply count n-gram overlap between generated and reference text—are known to poorly correlate with human evaluations of natural language generations^83–85^. We also lack automated metrics for evaluating the more qualitative aspects of a model’s “alignment” with human values (e.g., helpfulness or harmlessness)^86^, even as the importance of human feedback during training has been repeatedly demonstrated via techniques like Reinforcement Learning from Human Feedback^87^. This is especially worrying in medical settings, where patient safety is paramount. CLaMs that might impact clinical decisions should be evaluated much more rigorously than automated metrics can provide, across axes such as agreement with scientific consensus, minimization of the extent and risk of harm, possibility of bias, and the clinical utility of the advice^29,82^.
2. **Less labeled data:** The simplest way for researchers to demonstrate how clinical FMs exhibit improved sample efficiency is to replace evaluation metric “*X”* with the more nuanced metric “*X using K training examples”*. For example, replacing “*AUROC*” with “*AUROC using 1000 labeled radiology reports for fine-tuning.”* Ideally, a clinical FM would enable similar model performance at low values of *K* as at high values of *K*. Another way to demonstrate improved sample efficiency is to measure *zero-shot* and *few-shot* model performance, in which a model is given either zero or <100 examples, respectively, for the task on which it is evaluated. Researchers should also consider measuring the performance difference between fine-tuning versus prompting, where the former has been known to achieve higher accuracy, but the latter represents a much simpler and more flexible deployment option (as the model weights remain frozen)^88^. One could also measure the *total dataset annotation time saved* by using a clinical FM, measured in terms of dollars or hours.
3. **Simplified model deployment:** To quantify the value of FMs in lowering the barrier for building task-specific models^1,89^, one possible metric is the cost of hardware/compute/memory needed to train a model or generate a prediction. More broadly, we can measure the overall cost savings of using a clinical FM in terms of full-time equivalents (FTEs) or resource hours saved when downstream models (e.g., risk of inpatient mortality) are built on top of a clinical FM versus training a task-specific model from scratch. We recognize, however, that this evaluation may be the most challenging to conduct, as it requires buy-in from the business, clinical, and IT units of a health system. Health systems with dedicated ML Operations (“MLOps”) teams may be better positioned to realize these benefits^90^.
4. **Emergent clinical applications:** Clinical FMs can perform entirely novel tasks thought to be beyond the reach of machines even just a year ago, e.g., summarizing MRI reports in patient-accessible terms, writing discharge instructions, or generating differential diagnoses^45,91^. “Emergence” is a term of art used by ML researchers to describe the phenomenon by which FMs trained on large datasets are able to perform tasks that were impossible for smaller ML models to accomplish^7^. While this greatly broadens the range of clinical problems addressable via machine learning, it is still unproven whether these capabilities provide tangible utility to health systems in production settings^92^. Thus, we must explicitly define the scenarios in which the emergent capabilities of clinical FMs achieve their purported benefits. For example, LLMs such as GPT-4 can produce new USMLE exam questions, which are indistinguishable from human-authored questions. However, whether the use of these questions results in better-prepared medical students, or a lower burden for creating exam questions, remains to be quantified^93^.
5. **Multimodality:** Currently, the majority of evaluation tasks span one data modality^78^, even though models that simultaneously use multiple data modalities show substantial gains^94^. There is a strong unmet need for evaluation scenarios which explicitly require multimodal representations. Many datasets already include multimodal data (e.g., MIMIC-III, eICU, private EMRs, etc.), but evaluation tasks are not constructed in ways that require the demonstration of multimodal reasoning across both structured data and unstructured text. A great example of datasets that accomplish this are the Holistic AI Framework (HAIM), which builds on top of MIMIC-III to enable truly multimodal evaluation scenarios^95^.
6. **Novel human-AI interfaces:** Human evaluation and usability studies are needed to quantify the utility of interacting with FMs via prompts^1^. Metrics include user satisfaction, engagement, system usability scale scores, qualitative interview feedback, and the time/effort required to achieve stated goals^96–98^. Measuring the skill level necessary to operate a model can also shed light on its ability to empower providers to perform a multitude of roles. For FEMRs, an accepted paradigm for “prompting” does not yet exist, so developing a framework for prompting a patient’s medical history would represent a significant step forward. One exception is the Clinical Decision Transformer, which used a desired clinical end state (e.g., normal A1C levels) as a prompt to generate medication recommendations^55^.

## Discussion

Our review of 50 CLaMs and 34 FEMRs shows that most clinical FMs are being evaluated on tasks that provide little information on the potential advantages of FMs over traditional ML models. While there is ample evidence that clinical FMs enable more accurate model predictions, Figs. 2, 3 show that minimal work has been conducted to validate whether the other, potentially more valuable benefits of FMs will be realized in healthcare. These benefits include reducing the burden of labeling data, offering novel human-AI interfaces, and enabling new clinical applications beyond the reach of traditional ML models, among others outlined in “Benefits of Clinical FMs”. To help bridge this divide, we advocate for the development of new evaluation tasks, metrics, and datasets more directly tied to clinical utility, as summarized in Fig. 4.

While we focused this review on FMs developed specifically for clinical data, we recognize that there has been significant recent progress in adapting general-purpose LLMs to medical knowledge tasks^11^. As these general-purpose models continue to improve, the need and value of having clinical-specific models remain an open question^32^. However, it is worth emphasizing that the evaluation of these general-purpose LLMs suffers from the same exact limitations as evaluations of clinical LLMs, and the critiques described in this review still apply. While general-purpose LLMs continue to improve on specific clinical tasks, e.g., clinical knowledge and board certification benchmarks, it remains unclear how well they perform for broader applications in the hospital and what is achievable without training on some degree of in-domain data (e.g., EHRs). For example, the fact that GPT-4 passes the USMLE does not necessarily mean the model is useful for the types of questions clinicians care about in practice^82^. We believe more work needs to be done to assess the clinical reasoning capabilities of these general-purpose systems, and to develop a better theoretical understanding of how a model’s skills in other domains strengthen or worsen its performance on clinical tasks. There are also considerations beyond overall accuracy, such as scalability and inference cost, that may have different trade-offs in smaller, more targeted clinical-specific FM deployments^99^.

In addition to the potential benefits listed in “What are Clinical FMs?”, FMs present numerous risks that must also be considered and investigated. Data privacy and security are significant concerns with FMs, as they may leak protected health information through model weights or prompt injection attacks^100,101^. FMs are also more difficult to interpret, edit, and control due to their immense size^102^. They require high up-front costs to create, and while these costs can be amortized over multiple downstream applications, their value may take longer to realize than a smaller model developed for a single high-value task^103^. Additionally, FMs may fall under Software-as-a-Medical-Device guidelines regulating their usage in the clinic^104^. And similar to traditional ML models, FMs are susceptible to biases induced by miscalibration or overfitting^105^, as well as inducing “automation bias” in which clinicians defer to a model’s outputs even when they are obviously incorrect^106^. Developing frameworks for determining a model’s overall worth remains indispensable^79^.

Despite these challenges, clinical FMs hold immense promise for solving a diverse range of healthcare problems. We invite the research community to develop better evaluations to help realize their potential for benefiting both patients and providers^22^.

## Data Availability

We do not have any data beyond what is depicted in the Figures of this paper.
